# The Activity of Menkes Disease Protein ATP7A Is Essential for Redox Balance in Mitochondria*

**DOI:** 10.1074/jbc.M116.727248

**Published:** 2016-05-16

**Authors:** Ashima Bhattacharjee, Haojun Yang, Megan Duffy, Emily Robinson, Arianrhod Conrad-Antoville, Ya-Wen Lu, Tony Capps, Lelita Braiterman, Michael Wolfgang, Michael P. Murphy, Ling Yi, Stephen G. Kaler, Svetlana Lutsenko, Martina Ralle

**Affiliations:** From the Departments of ‡ Physiology,; ¶ Biological Chemistry, and; ‖ Cell Biology Johns Hopkins University School of Medicine, Baltimore, Maryland 21205,; the § Department of Molecular and Medical Genetics, Oregon Health and Science University, Portland, Oregon 97239,; the ** Medical Research Council Mitochondrial Biology Unit, Cambridge CB2 0XY, United Kingdom, and; the ‡‡ Section on Translational Neuroscience, Eunice Kennedy Shriver National Institute of Child Health and Human Development, National Institutes of Health, Bethesda, Maryland 20892

**Keywords:** biosensor, copper transport, mitochondria, neurological disease, oxidation-reduction (redox), ATP7A, Menkes disease, glutathione

## Abstract

Copper-transporting ATPase ATP7A is essential for mammalian copper homeostasis. Loss of ATP7A activity is associated with fatal Menkes disease and various other pathologies. In cells, ATP7A inactivation disrupts copper transport from the cytosol into the secretory pathway. Using fibroblasts from Menkes disease patients and mouse 3T3-L1 cells with a CRISPR/Cas9-inactivated ATP7A, we demonstrate that ATP7A dysfunction is also damaging to mitochondrial redox balance. In these cells, copper accumulates in nuclei, cytosol, and mitochondria, causing distinct changes in their redox environment. Quantitative imaging of live cells using GRX1-roGFP2 and HyPer sensors reveals highest glutathione oxidation and elevation of H_2_O_2_ in mitochondria, whereas the redox environment of nuclei and the cytosol is much less affected. Decreasing the H_2_O_2_ levels in mitochondria with MitoQ does not prevent glutathione oxidation; *i.e.* elevated copper and not H_2_O_2_ is a primary cause of glutathione oxidation. Redox misbalance does not significantly affect mitochondrion morphology or the activity of respiratory complex IV but markedly increases cell sensitivity to even mild glutathione depletion, resulting in loss of cell viability. Thus, ATP7A activity protects mitochondria from excessive copper entry, which is deleterious to redox buffers. Mitochondrial redox misbalance could significantly contribute to pathologies associated with ATP7A inactivation in tissues with paradoxical accumulation of copper (*i.e.* renal epithelia).

## Introduction

Copper is an essential cofactor for numerous enzymes, including Cu/Zn-superoxide dismutase (SOD1), cytochrome *c* oxidase (COX), tyrosinase, dopamine-β-hydroxylase, lysyl oxidase, and many others. These enzymes are involved in physiological processes that are indispensable for life. Consequently, copper deficiency is deleterious and can result in death (1, 2). Copper is transported into cells predominantly by a copper transporter, CTR1. This process is facilitated by intracellular glutathione (3). Excess copper is removed from the cell by the ATP-driven copper transporters (Cu(I)-ATPases) ATP7A and ATP7B. ATP7A is the major regulator of copper homeostasis in most human cells. ATP7A uses the energy of ATP hydrolysis to transfer copper from the cytosol into the lumen of secretory pathway for functional maturation of copper-dependent enzymes within this compartment. ATP7A also sequesters excess copper in vesicles, which eventually fuse with the plasma membrane, allowing copper export. Inactivation of ATP7A results in fatal Menkes disease (1, 2). ATP7A mutations have also been linked to occipital horn syndrome and isolated distal motor neuropathy (4). In these allelic variants, mutant ATP7A retains some function, and the diseases have a milder course with better survival. Several inbred mouse strains with mutations in ATP7A exist and have been used to explore the consequences of ATP7A inactivation (5–7). Recently, a targeted deletion of ATP7A in motor neurons in mice was shown to trigger age-dependent muscle atrophy resembling the phenotype of human X-linked spinal muscular atrophy type 3. In this latter case, the role of ATP7A in systemic copper homeostasis was unaltered, and the pathology reflected the loss of important ATP7A functions in motor neurons (8).

The functional significance of ATP7A at the level of the whole organism is firmly established. ATP7A facilitates export of copper from the intestine and mediates copper entry into the brain (9). ATP7A inactivation results in systemic copper deficiency, especially in the CNS. In brains of Menkes disease (MD)^6^ patients, activity of copper-dependent enzymes is decreased, and changes in myelination, energy metabolism, catecholamine balance, and mRNA translation are evident (10). However, in MD, not all tissues are copper-deficient. Certain organs and tissues, *e.g.* the kidney and intestine, accumulate copper (11–13). In such peripheral tissues, the mechanisms of pathology caused by ATP7A inactivation may not be identical to those in the CNS (4–7). Copper supplementation therapy, commonly used to improve conditions in MD, may exacerbate the copper-accumulating tendency in such tissues and have a negative impact (13, 14).

Currently, information about the cellular consequences of ATP7A inactivation is limited. Studies using patient skin fibroblasts have shown that loss of ATP7A function results in elevation of cellular copper content (4, 5) and up-regulation of proteins involved in copper sequestration (metallothioneins) and DNA repair (15). It remains unclear whether copper acts primarily in the nuclei or whether detected changes in the mRNA profiles are caused by metabolic changes in other compartments and/or intercompartment signaling (6). It was proposed that mitochondria contribute to the maintenance of cellular copper balance by communicating changes in its metabolic status to ATP7A (16). Whether and how inactivation of ATP7A alters the functions of mitochondria or any other cell compartment beyond the secretory pathway is unclear. In this study, we have systematically addressed this issue.

We have found that, in ATP7A^−/−^ cells (human skin fibroblasts as well as mouse preadipocytes), copper is elevated in several intracellular compartments and has distinct effects on their redox environment. Mitochondria are most impacted and show increased oxidation of glutathione and thioredoxin (the two major redox buffers) as well as accumulation of peroxide. Although cell growth is not inhibited, ATP7A^−/−^ cells are highly sensitive to a decrease of total glutathione content. These results highlight a previously unanticipated consequence of ATP7A dysfunction for mitochondrial redox homeostasis and suggest that abnormal mitochondrial redox status may contribute significantly to MD pathogenesis in peripheral tissues and to pathologies associated with a cell-specific loss of ATP7A function. Cells and tissues with a low glutathione content and/or high demand for mitochondrial function may be especially at risk.

## Experimental Procedures

### 

#### 

##### Cell Culture and Fractionation

Generation of immortalized skin fibroblast from an MD patient (YS cells) and the mother of this patient (XS cells) were described previously (17)). The mutation in ATP7A in YS cells, a single base pair deletion of cytidine1638 in exon 6, was identified using exon amplification and direct sequencing of the PCR products. A detected deletion of a base pair in nucleotide C1638 in the patient ATP7A transcript is predicted to produce a frameshift at amino acid position 546, followed by eight unrelated amino acids. XS cells showed the same PCR product but uniformly lacked the mutation; *i.e.* the genotype of control cells was ATP7A^+/+^. The lack of mutation is due to the mosaic nature of the skin of the carrier, which permits isolation of cells with both normal alleles. The control fibroblast cell line GM3440 was derived from an unrelated healthy donor. Two MD fibroblast cell lines (MF1 and MF2) were derived from a patient with mutation Q724H (18) and IVS8 AS dup5 mutation (19), respectively. The *ATP7A*^−/−^ *3T3-L1* cells were generated as described below using mouse 3T3-L1 cells (ATCC) and the CRISPR/Cas9 protocol.

The fibroblasts and HEK293T and 3T3-L1 cells were cultured in DMEM (Gibco) supplemented with 10% FBS (Sigma-Aldrich) and 1% Penstrep (Gibco). For subcellular fractionation experiments, cells grown on 15-cm dishes were washed with PBS, trypsinized, and washed with PBS. Cell pellets were resuspended with 1 ml of lysis buffer (PBS containing 1 mm EDTA, 1 mm EGTA, 1 mm 4-(2-aminoethyl)benzenesulfonyl fluoride hydrochloride, 0.1 volume of 2.5 m sucrose, and protease inhibitor mixture (Roche)), and homogenized with a loose pestle Dounce homogenizer (100 strokes) and 220 strokes with a tight pestle (step 1). The homogenate was centrifuged at 600 × *g* for 6 min. The pellet was washed twice in PSE buffer (PBS containing 1 mm EDTA, 300 mm sucrose, 0.1% IGEPAL, and complete protease inhibitor mixture (Roche)). The pellet was resuspended in PSE buffer containing 0.2% Triton X-100 and used as a nucleus-enriched fraction. The supernatant from step 1 was centrifuged at 10,000 × *g* for 10 min to pellet mitochondria. The pellet was resuspended in PSE buffer containing 0.2% Triton X-100. The supernatant was kept as a post-mitochondrial fraction (cytosol). Mitochondrion purity was examined by Western blotting (*n* = 3) and densitometry using lamin and TIM23 as markers of nuclei and mitochondria, respectively. The nuclear contamination of the mitochondrial fraction was 24%.

##### Copper Content in Cells and Cell Fractions

Cells were transferred to a precleaned polypropylene tube and pelleted by centrifugation, and the resulting pellets were washed with ice-cold PBS. Cell pellets and cell fractions were digested in 50% HNO_3_ (trace metal-grade, Fisher Scientific, Fair Lawn, NJ) by incubating at 90 °C for 3 h. Upon completion, an equal amount of hydrogen peroxide (trace metal-grade, Fisher Scientific) was added, and the resulting solution was diluted with 1% HNO_3_ to achieve a final concentration of HNO_3_ below 5%. Inductively coupled plasma (ICP) MS analysis was performed using an Agilent 7700X instrument equipped with an ASX 500 autosampler. The system was operated at a radio frequency power of 1550 W, an argon plasma gas flow rate of 15 liters/min, and an argon carrier gas flow rate of 1.04 liters/min. Elements were measured in kinetic energy discrimination mode using helium gas (4.3 ml/min). Data were quantified using a 10-point calibration curve (0.5–5000 ppb (ng/g) range) with external standards for calcium, manganese, iron, copper, and zinc (elemental mix 2, VHG Labs, Manchester, NH) in inorganic diluent (1% HNO_3_) to match the sample matrix. For each sample, data were acquired in triplicate and averaged. Internal standards (^45^Sc, ^72^Ge, and ^209^Bi) were introduced with the sample to correct for plasma instabilities. A 10-ppb standard solution containing calcium, manganese, iron, copper, and zinc and a blank containing diluent only were used to calculate the coefficient of variance. To ensure maximum recovery of elements, certified National Institute of Standards and Technology (Gaithersburg, MD) standard reference material (bovine liver, SRM 1577c) was treated and prepared by the same method as the samples and analyzed. To determine background concentrations from materials used in the analysis, a blank tube was treated as a sample, and elemental contents were measured. All glass- and plasticware used in ICP-MS analysis was rinsed with and/or stored in 1% HNO_3_ until use. To quantify the concentration of elements in cells, the diameters of trypsinized cells (which have a spherical shape) were determined using the automated features of a cell counter (TC10, Bio-Rad) and averaged and cell volumes were calculated.

The copper content of control, MF1, MF2, and ATP7A^−/−^ 3T3-L1 cells was determined using a Shimadzu 6650 graphite furnace atomic absorption spectrophotometer equipped with an ASC-6100 autosampler. Cell pellets were dissolved with HNO_3_ and heated at 50 °C for 30 min. Samples were diluted with double-distilled H_2_O to be in the linear absorption range of the calibration curve (1–10 ppb). Each sample was measured two to three times, and the concentration of copper was derived from the calibration curve. Protein concentration or initial cell number was used for normalization. Experiments were repeated three times with three technical replicates.

##### X-ray Fluorescence Microscopy

For x-ray fluorescence microscopy experiments, cells were grown on 4 × 4 mm silicon nitride membranes (Silson Ltd., North Hampton, England). Silicon nitride membranes were sterilized with UV radiation and incubated with 10 μl of sterile 0.01% poly-l-lysine solution (Sigma-Aldrich, St. Louis, MO) at 37 °C. After 30 min, poly-l-lysine was removed, and 10 μl of cell-containing medium was added to the membrane. Basal medium was added to the cultures after 15 min at 37 °C. After completion of the experiments, the membranes were rinsed with PBS; fixed with 2% paraformaldehyde for 30 min at 37 °C; rinsed with PBS, isotonic 100 mm ammonium acetate, and deionized water; and air-dried. X-ray fluorescence microscopy data were collected on beamline 2-ID-E at the Advanced Photon Source (Argonne National Laboratory, Argonne, IL). Silicon nitride membranes were mounted on kinematic sample holders, and target cells were selected using a light microscope (Leica, Buffalo Grove, IL) equipped with a high-precision, motorized x-y stage (Ludl Electronic Products, Hawthorne, NY). The coordinates of target cells were recorded before mounting the sample on the microprobe stage at the beamline. The microscope coordinates were translated into microprobe coordinates, and the cell raster was scanned in the x-y plane. The incident x-ray energy was tuned to 10 keV using a Si monochromator. The monochromatic beam was focused to 750 × 750 nm using a Fresnel zone plate. The sample was placed at 19° to the incident x-ray beam, and the resulting x-ray fluorescence was collected at 90° using an energy dispersive, four-element detector (Vortex ME-4, SII Nanotechnology, Northridge, CA). Elemental maps were created by extracting, background-subtracting, and fitting the fluorescence counts for each element at each point using the program MAPS (20). The fluorescent photon counts were translated into micrograms per square centimeter using calibrated x-ray standards (AXO Products, Dresden, Germany).

##### Generation of NLS-GRX1-roGFP2 and MTS-GRX1-roGFP2

To target the sensor to the nucleus, the SPKKKRKVE nuclear localization signal (NLS) from SV40 and a linker sequence (SG)^4+4^ were added at to the N terminus of GRX1-roGFP2 (21). The DNA sequence 5′-AGCCCGAAGAAAAAACGTAAAGTGGAG-3′ encoding the NLS and the 5′-AGCGGGAGC-GGGAGCGGGAGCGGG-3′ sequence encoding the linker were inserted at the 5′ end of GRX1-roGFP2 cDNA using overlapping PCR. The insert was cloned into the pEIGW vector using SmaI and EcoRI restriction sites at the 5′ and 3′ end, respectively. To target a sensor to mitochondria (MTS-GRX1-roGFP2), three mitochondrial target sequences (MTSs) MSVLTPLLLRGL-TGSARR were inserted in tandem at the N terminus of GRX1-roGFP2. This was done by inserting three copies of the 5′ ATGTCCGTCCTGACG-CCGCTGCTGCTGCGGGGCTTGACAGGCTCGGCCCGGCGG 3′ sequence at the 5′ end of GRX1-roGFP2 cDNA using overlapping PCR.

##### Live Cell Imaging

To evaluate the GSH:GSSG ratio, the YS and control cells were plated on FD-35 Fluorodishes and transiently transfected with 1000 ng of GRX1-roGFP2, NNLS-GRX1-roGFP2, or MTS-GRX1-roGFP2 using Lipofectamine LTX Plus (Life Technologies) following the protocol of the manufacturer. Cells were cultured overnight in DMEM without phenol red (Gibco) and then imaged with a Zeiss LSM510 Meta and Zeiss LSM700 confocal laser scanning microscope. The sensor was excited frame by frame with a 405- and 488-nm laser, and emission was detected using a 500–554 band pass filter. To verify the responsiveness of the GRX1-roGFP2 sensors to oxidation/reduction, HEK293T cells transiently expressing the sensors were imaged under basal conditions and in response to treatment with 100 μm H_2_O_2_ and 500 μm DTT. Images showing the ratio of fluorescence intensities after exciting at two wavelengths (IR_405/488_) were prepared using Zen2009 software after subtracting the background. To measure the levels of peroxide YS, control and HEK293T cells were transiently transfected with the HyPer sensor targeted to the nucleus, mitochondria, or cytosol (22). Intensity ratio (IR_488/405_) images were also prepared using Zen2009 software after subtracting the background.

##### Analysis of Glutathione in Cell Lysates and Fractions

All reactions and dilutions, unless mentioned otherwise, were conducted in 10 mm sodium phosphate and 1 mm EDTA (pH 7.2). 400 μl of 5% 5-sulfosalicylic acid was added to each cell pellet (∼1.6 w/v dilution) to lyse cells and deproteinize cell contents. The lysis product was stored at −80 °C until use and spun down. The supernatant was used to determine soluble GSH. The deproteinization step was avoided in the estimation of GSH in organelle extracts. For copper chelation, the mitochondrion-enriched fraction was incubated with 50 or 100 μm bathocuproine disulfonate on ice for 3 h. Glutathione content was determined by a modified version of the colorimetric recycling assay using 5,5′-dithiobis(2-nitrobenzoic acid) (DTNB) (23). Absorbance of the reaction product was monitored at 412 nm for up to 40 cycles using a microplate reader (FLUOstar Omega, BMG Labtech). The slope was determined using the linear range of the curve and compared with that of a standard curve for known GSH concentrations. All samples were assayed in triplicate, and the results were corrected from the total cell count and volume for a given sample.

##### Rescue of Mitochondrial Glutathione Balance by Restoration of Copper Export

Several attempts to generate human Cherry-ATP7A were unsuccessful because of rearrangements within the cDNA sequence. Consequently, to restore copper transport within the secretory pathway, a homologous ATP7B was used. YS cells were cultured on Fluorodishes and transiently cotransfected with the Cherry-ATP7B and MTS-GRX1-roGFP2 plasmids (1000 ng of each), following the protocol described above. Cells co-expressing proteins and those expressing only MTS-GRX1-roGFP2 were selected for comparison from the same culture dish.

##### Electron Microscopy

MF1, MF2, and control GM3440 cells were fixed in 2% glutaraldehyde, 2% paraformaldehyde, 0.1 m sodium cacodylate, and 3 mm MgCl_2_ (pH 7.2) for 1 h at room temperature. After a buffer rinse, the samples were incubated for 1.5 h in 1% osmium tetroxide in 0.1 m sodium cacodylate and 3 mm MgCl_2_ on ice in the dark. Following a wash with 50 mm maleic acid and 3% sucrose (pH 6.2), samples were stained with 2% uranyl acetate for 1 h at room temperature in the dark. Samples were dehydrated in a graded series of ethanol and embedded in Eponate 12 (Ted Pella) resin. Samples were polymerized at 37 °C for 2–3 days before moving to 60 °C overnight. Thin sections, 60–90 nm, were cut with a diamond knife on a Reichert-Jung Ultracut E ultramicrotome and placed on 2 × 1 mm formvar-coated copper slot grids, and the grids were stained with 2% uranyl acetate in 50% methanol and 0.4% lead citrate (prepared by Johns Hopkins University School of Medicine MicFac). The sections were then examined on a Hitachi 7600 transmission electron microscope at 80 kV. Images were captured with an Advanced Microscopy Techniques XR50 CCD (5-megapixel) camera.

##### Cytochrome c Oxidase Activity

YS and control cells were grown on six 15-cm dishes to 80–90% confluence. Cell pellets were resuspended in IBc (10 mm Tris, EGTA, and 200 mm sucrose) buffer and homogenized with 45 strokes in a precooled small Teflon Dounce at 1600 rpm. The homogenate was centrifuged twice at 600 × *g* for 10 min at 4 °C. The supernatant was collected and centrifuged at 7000 × *g* for 10 min at 4 °C. The pellet was washed by resuspending in IBc buffer and centrifuging at 7000 × *g* at 4 °C. The pellet was resuspended in IBc buffer and centrifuged at 10,000 × *g* for 10 min at 4 °C and resuspended in 250 mm mannitol, 5 mm HEPES (pH 7.4), and 0.5 mm EGTA.

The activity of complex IV was measured by following the initial rate of ferricytochrome *c* oxidation at 550 nm using a protocol adapted from Ref. 7. Briefly, equine cytochrome *c* at 0.8% (weight per reaction volume of 50 mm potassium phosphate, 2 mm EDTA (pH 7.4) reaction buffer) was reduced with 1 m DTT (0.1 μl/80 ml of solution) for 20 min at room temperature. To decrease concentration of DTT, the cytochrome *c* solution was then concentrated using Amicon Ultra 0.5-ml 10K cutoff filters (Millipore), diluted with buffer to a final concentration of 0.08% (w/v), and then with water to reach an optical density of 1.8–1.9. The reaction was initiated by addition of 30 μg of mitochondrial fraction solubilized with 0.5% *n*-dodecyl β-d-maltoside (Anatrace) at a final concentration of 5 mg/ml in reaction buffer supplemented with 1 mm PMSF, 10 μm leupeptin, and 2 μm pepstatin A, and the change in absorbance was followed for 150 s. Each sample was measured three times, and the data were averaged. The rate of oxidation was expressed as a change in cytochrome absorption per minute divided e_550_ × mg of mitochondria, where e_550_ = 19.6 mmol × L^−1^ × cm^−1^.

##### Oxidation State of Thioredoxin (Trx1)

Differentially oxidized forms of Trx1 were characterized as described previously (24, 25). Briefly, cells grown in basal medium were harvested, resuspended in a buffer containing 6 m guanidine, 50 mm Tris-HCl (pH 8.3), and 3 mm EDTA and sonicated for 4 s four times followed by centrifugation at 1000 × *g* and incubated with 5.5 μm iodoacetic acid at 37 °C for 30 min to carboxymethylate Trx1. To generate fully reduced and oxidized samples, samples were treated for 30 min with either 40 mm DTT or 5 mm H_2_O_2_, respectively, prior to iodoacetic acid incubation. Gel electrophoresis was performed using a 12% native precast polyacrylamide gel and native Tris/glycine buffer at 225 V on ice. Proteins were transferred to a PDVF membrane and probed using rabbit anti-Trx1 antibody (1:500 in 5% milk Tris-buffered saline with 1% Triton X-100, overnight at 4 °C, Santa Cruz Biotechnology) and horseradish peroxidase-conjugated goat anti-rabbit secondary antibody (1:10,000 in 5% milk TBST, 1.5 h, Abcam), followed by chemiluminescent detection with ECL Western blotting substrate (Pierce, Thermo Scientific) using a FluorChem E system (Protein Simple).

##### Generation of 3T3-L1-ATP7A^−/−^ Cells Using the Cas9-CRISPR System

The human codon-optimized Cas9 expression vector was obtained from Addgene (plasmid 41815) (26), and Cas9 was cloned into a pEF6 expression vector downstream and in-frame with a nuclearly localized YFP linked by a viral 2A bicistronic peptide (27) so that nls-YFP and Cas9 were expressed in approximate equimolar quantities. A guide RNA, including the U6 promoter, was synthesized as a 500-bp gBlocks fragment (Integrated DNA Technologies) and was cloned into the pEF6-nls-YFP-2A-Cas9 vector by InFusion Cloning (Clontech). The single-guide RNA (sgRNA) sequences were designed to target trans-membrane regions of the ATP7A gene and determined by the CRISPR Design Tool. Two synthesized single-guide RNA oligos (Integrated DNA Technology) for each gene (ATP7A target 1, GTTTTTCTGTATCCCTGTAATGG; ATP7A target 2, CCTATGCTGTTTGTGTTTATTGC) were ligated into the Cas9 vector using the In-Fusion HD cloning kit (Clontech Laboratories). Undifferentiated 3T3-L1 cells were transfected with the resulting plasmid using Lipofectamine LTX Plus reagent (Life Technologies). Transfected cells were selected by 3 μg/ml blasticidin in cell culture medium for 2 weeks and then diluted into a 96-well plate for cell cloning. The clones were expanded into larger plates until the cell number exceeded 1 × 10^6^, and the cryostocks were prepared. 3T3-L1-ATP7A^−/−^ cells were identified by Western blotting analysis of cell homogenates. Briefly, cells were collected from a 10-cm dish and homogenized in 25 mm imidazole (pH 7.4), 0.25 m sucrose, 2 mm 4-(2-aminoethyl) benzenesulfonyl fluoride hydrochloride (AEBSF), and one tablet of EDTA-free protease inhibitor mixture. The protein concentration was estimated using a BCA assay. 20 μg of homogenate was separated on 7.5% polyacrylamide gels, transferred onto a PVDF membrane, and incubated overnight at 4 °C with rabbit anti-ATP7A CT77 (Hycult Biotech) to detect ATP7A and with mouse anti-tubulin (Sigma), used as a loading control. Deletions in the ATP7A gene were identified by Sanger sequencing. Specifically, genomic DNA from positive cell clones was isolated using the GenElute mammalian genomic DNA miniprep kit (Sigma-Aldrich), and the targeted region of the ATP7A gene was amplified by PCR using the forward primer 5′-TCTTAGCCTGAGTGAGATGGTT-3′ and the reverse primer 5′-TCCACTATCTTAACAA-ATGTCACCC-3′. PCR products were cloned into the TOPO vector using the TOPO TA cloning kit (Life Technologies) and transformed into Top 10-competent cells (Life Technologies). Plasmids were then isolated using the QIAprep spin miniprep kit (Qiagen) for Sanger sequencing.

##### Redox Imaging

Control, MF1, MF2, and 3T3-L1-ATP7A^−/−^ cells transiently expressing MTS-GRX1-roGFP2 were grown in 35-mm dishes and treated with 200 μm H_2_O_2_ or 1 mm DTT to achieve complete sensor oxidation and reduction, respectively. Ratiometric (IR_405/488_) images were prepared using ImageJ software after subtracting the background. IR_405/488_images were then converted to percentage oxidation of MTS-GRX-roGFP2 using Hanson's equation.

##### Real-time PCR

To compare the levels of IFI27 mRNA, YS and control cells were cultured in 6-cm tissue culture dishes and treated with either 0 or 50 nm MitoQ for 24 h. For relative quantitation of Metallothionein 1 (MT1) mRNA, YS and control cells were cultured under basal conditions. Cells were then harvested, washed with PBS, and lysed using QIAshredder (Qiagen), and RNA was extracted with the RNeasy mini kit (Qiagen). The cDNA was prepared by reverse transcription using the Transcriptor first strand cDNA synthesis kit (Roche Applied Science) using 1 μg of RNA in a final volume of 20 μl using both anchored oligo(dT)18 and a random hexamer primer following the instructions of the manufacturer. Real-time PCR was performed using 1 μl of the prepared cDNA in a 20-μl reaction volume with Power SYBR Green PCR Master Mix (Applied Biosystems). The levels of IFI27 transcript were estimated using primers against a region of cDNA common for IFI27 isoforms. Forward primer 5′ CTCAGGAACTCTCCTTCTTTGG 3′ (exon 3) and reverse primer 5′ CCTGCTCGGGTTA-ATTCCGTGG 3′ were used. The amount of MT1 transcript was estimated using forward primer 5′ CCTGTGCCAGCTCCTGCAAGTGC 3′ and reverse primer 5′ CCAGGTTTGTGCAGG-TTGCTCTG 3′. β-Actin mRNA was used as a reference. The PCR involved initial denaturation at 95 °C for 10 min, 40 cycles of 15 s of melting at 95 °C, and 1-min annealing/extension at 60 °C. Relative mRNA abundance was quantified using the 2^−ΔΔCT^ method. Each sample was analyzed three times, and the data were averaged.

##### Glutathione Depletion and Cell Survival Assay

YS and control cells were plated into a 96-well plate at 2000–3000 cells/well. Cells were kept overnight at 37 °C and 5% CO_2_ to allow attachment. Cells were treated with 0–100 μm BSO and incubated for 48 h at 37 °C and 5% CO_2_. The BSO solution was renewed after 24 h. Cells were washed after 48 h with PBS and incubated with 120 μl of solution containing 25 μm phenazine methosulfate and 333 μg/ml 3-(4,5-dimethylthiazol-2-yl)-5-(3-carboxy-methoxy-phenyl)-2-(4-sulfophenyl)-2H-tetrazolium in DMEM without FBS. Cells were incubated for 20 min and lysed with 50 μl of 10% SDS, and absorbance was recorded at 490 nm. Samples were measured in triplicates.

##### Statistical Analyses

Statistical analyses (comparisons between the experimental groups) were performed using Student's *t* test (two-tailed, unpaired). *p* <0.05 was considered to be statistically significant.

## Results

### 

#### 

##### Copper Is Elevated in the Intracellular Compartments of ATP7A^−/−^ Cells

**FIGURE 1.**
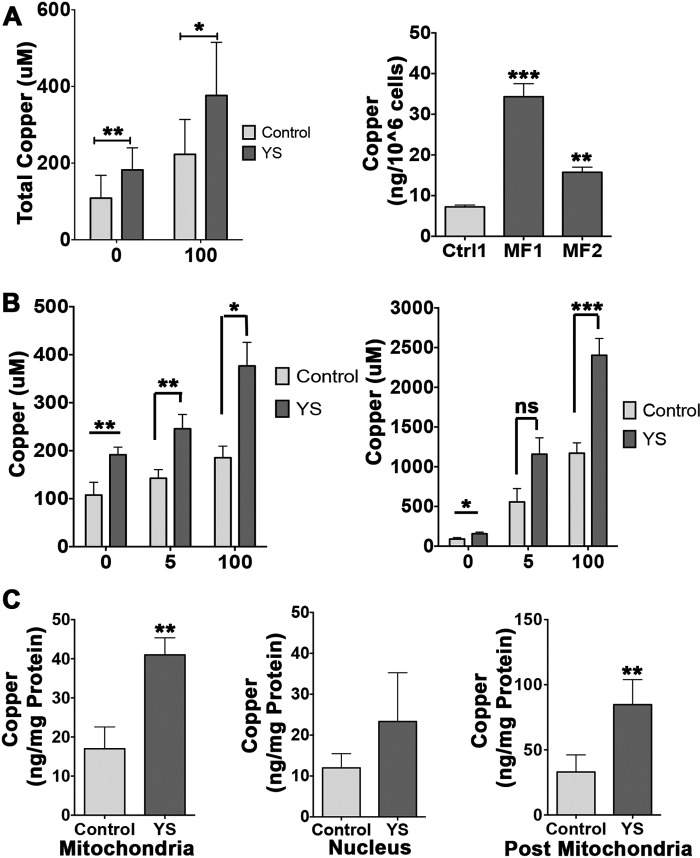
**Copper redistribution in control and MND patient cells.** *A*, *left panel*, copper levels in cells from the mother of the patient (*Control*) and the MD patient (*YS*) under basal conditions (108.7 ± 59.6 μm and 182.4 ± 57.4 μm, respectively; **, *p* = 0.0067) and in response to treatment with 100 μm CuCl_2_ for 4 h (222.8 ± 91.3 μm and 376.7 ± 138.5 μm, respectively; *, *p* = 0.0290). *Right panel*, copper levels under basal conditions in cells from an independent control/unrelated male (7.24 ± 0.78 ng/10^6^ cells) and an MD patient (MF1–34.33 ± 5.52 ng/10^6^ cells, MF2–15.7 ± 2.17 ng/10^6^ cells; ***, *p* = 0.0005; **, *p* = 0.0031). *Ctrl*, control. *B*, MD cells accumulate more copper in response to treatment with low (5 μm) or high (100 μm) CuCl_2_ compared with control cells. *Left panel*, copper levels in YS cells and control cells in basal medium (191.60 ± 15.86 μm and 107.56 ± 26.68 μm, respectively; **, *p* = 0.009), low copper (245.70 ± 29.87 μm and 142.70 ± 17.98 μm, respectively; **, *p* = 0.008), and high copper (376.76 ± 48.95 μm and 185.37 ± 24.09 μm, respectively; *, *p* = 0.03) after 4 h of treatment. *Right panel*, copper levels in YS and control cells in basal medium (156.43 ± 20.59 μm and 90.17 ± 18.07 μm, respectively; *, *p* = 0.03), low copper (1158.20 ± 205.90 μm and 557.20 ± 167.75 μm, respectively; *ns*, *p* = 0.15), and high copper (2403.65 ± 211.07 μm and 1171.35 ± 130.15 μm, respectively; ***, *p* = 0.0003) after 48 h of treatment. *C*, copper levels in subcellular fractions of control and YS cells. Mitochondria (17 ± 5.57 ng/mg protein and 41 ± 4.36 ng/mg protein in control and YS cells, respectively), nuclei (12 ± 3.46 ng/mg protein and 23.33 ± 11.93 ng/mg protein in control and YS cells, respectively), post-mitochondria −33 ± 13.11 ng/mg and 84.67 ± 19.22 ng/mg protein in control and YS cells, respectively). **, *p* = 0.0091; **, *p* = 0.0071. Data are mean ± S.D. of three independent experiments.

To understand the consequences of ATP7A inactivation, we used previously generated skin fibroblasts from MD patients, YS (immortalized), and MF1 and MF2 cells (18, 19), and control cells derived from the mother of one of the patients or an unrelated male. The main set of experiments was done using YS cells and control XS cells derived from the mother of a patient. MF1 and MF2 cells and an additional control cell line (from an unrelated male) were utilized to verify our main observations. Consistent with previous reports (28, 29), loss of ATP7A activity was associated with an increase in cellular copper content under basal growth conditions (Fig. 1*A*). Higher copper accumulation in patient cells compared with control cells was also evident following treatment of cells with low (5 μm) or high (100 μm) copper for 4 or 48 h (Fig. 1*B*, *left* and *right panels*, respectively).

**FIGURE 2.**
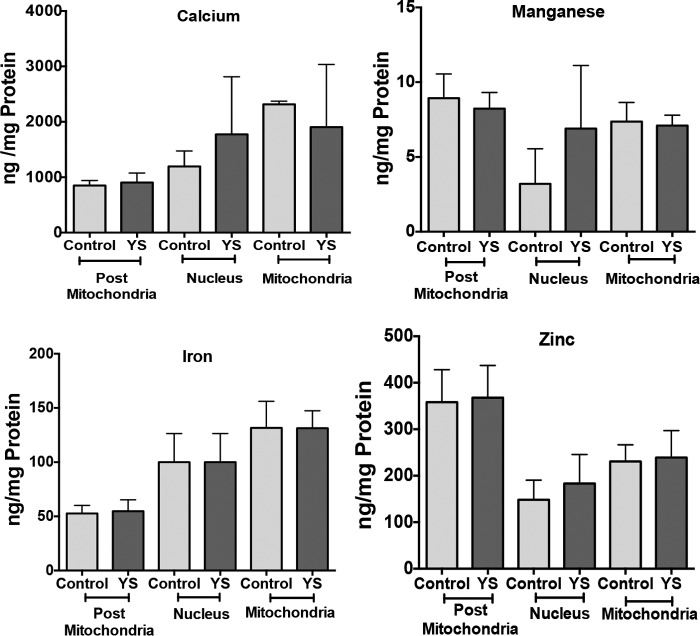
**The levels of metals other than copper are unchanged in the subcellular fractions of control and YS cells.** The values for manganese in nuclear extracts were variable (1.7–5.9 ng/mg protein for control cells and 3.8–11.7 ng/mg protein for YS cells) in contrast to other metals, which were measured in parallel using ICP-MS and yielded consistent values. Although there is an apparent trend to higher mean values in YS cells, the difference was not statistically significant. Mean ± S.D. from three independent experiments is shown.

Subcellular fractionation and ICP-MS analysis revealed that copper was elevated in all major intracellular compartments (Fig. 1*C*). In the nuclei, copper was higher on average, but the increase was not statistically significant. Mitochondria had a 2.4-fold increase of copper content, and the post-mitochondrial fraction, comprised of cytosol and microsomes, had a 2.6-fold higher copper content. Accumulation was specific for copper, with no changes detected for manganese, calcium, iron, and zinc (Fig. 2). Thus, excess cytosolic copper enters the membrane-encapsulated nuclei and mitochondria, where it remains sequestered during cell fractionation.

**FIGURE 3.**
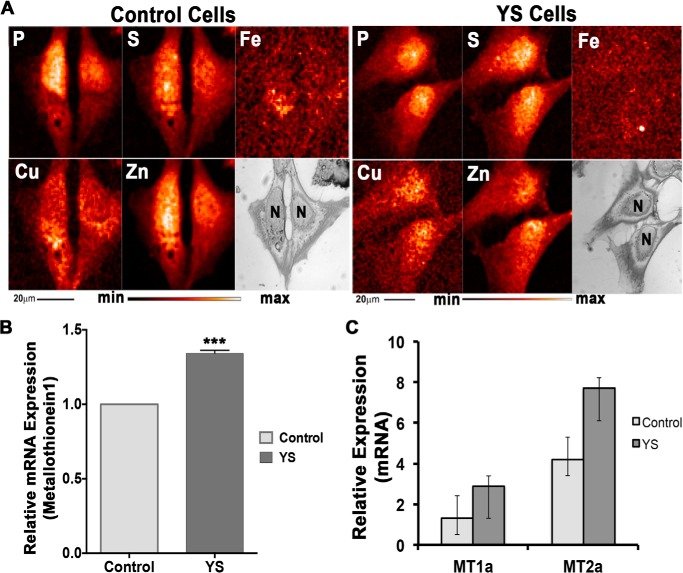
**Change in intracellular distribution of copper in YS cells is associated with up-regulation of metallothioneins.** *A*, x-ray fluorescence microscopy images for control and YS cells grown under basal conditions. Shown are visible light microscope images and elemental maps for phosphorus, sulfur, iron, copper, and zinc. The relative elemental concentration is presented by false coloring (*temperature bar*). *Scale bar* = 20 μm. *N*, nucleus. *B*, the relative expression of MT1 in YS cells is 1.35-fold higher than that in control cells under basal conditions. ***, *p* = 0.0011. *C*, increase in the levels of metallothionein (MT1 and MT2) transcripts in control and YS cells in response to treatment with 100 μm CuCl_2_ for 48 h. Values are given in comparison with basal conditions. Data are mean ± S.D. (*n* = 3).

The change in spatial distribution of copper was confirmed by x-ray fluorescence microscopy (Fig. 3). In control cells, copper was highest in the vicinity of nuclei but not in the nucleus, in agreement with results published previously (30). In patient cells, the pattern of copper distribution was altered. The perinuclear ring disappeared, and copper was distributed throughout the cell and enriched within nuclei (Fig. 3*A*). Higher copper content and changes in the intracellular distribution of copper in patient cells were associated with an increased expression of metallothioneins (MT1 and MT2) (Fig. 3*B*). Under basal culture conditions, the increase reflected moderate copper accumulation. Treatment with 5 or 100 μm copper for 48 h further increased the expression of MT1 and MT2, especially in YS cells, reflecting a higher copper load (Fig. 3*C*).

##### Probes for Quantitative Imaging of Glutathione Oxidation in Subcellular Compartments of Live Cells

**FIGURE 4.**
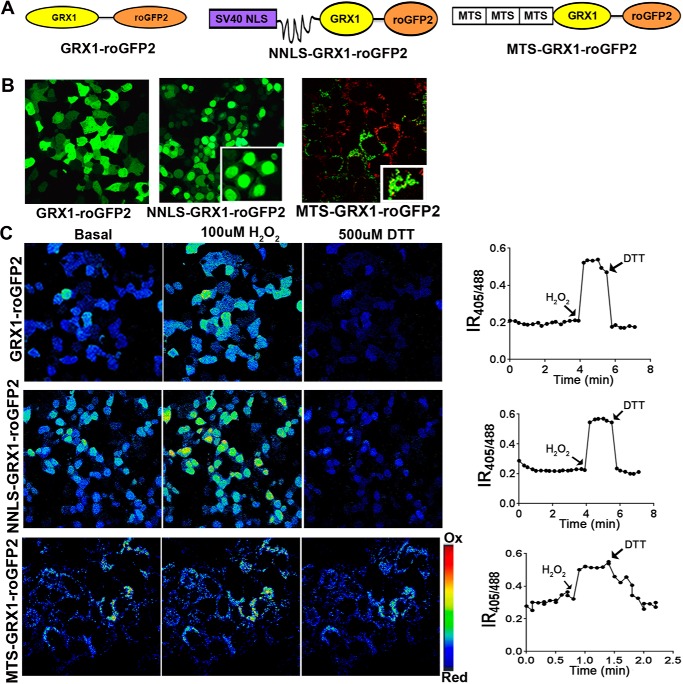
**Probes for measuring the GSH:GSSG ratio in cellular compartments.** *A*, schematic of the design of the GRX1-roGFP2-based sensors targeted to the cytosol (*GRX1-roGFP2*), nucleus (*NNLS-GRX1-roGFP2*), and mitochondria (*MTS-GRX1-roGFP2*). *B*, expression and subcellular localization of sensors. A diffuse pattern indicates cytosolic localization of GRX1-roGFP2. NNLS-GRX1-roGFP2 is targeted to nuclei. Targeting of MTS-GRX1-roGFP2 (*green*) to mitochondria is verified by colocalization with MitoTracker (*red*). *C*, *left panel*, ratiometric false color image of HEK293T cells expressing different sensors under basal conditions and their response to treatment with 100 μm H_2_O_2_ and 500 μm DTT. *Right panel*, representative changes in IR_405/488_ values in a single cell. *Arrows* indicate the time when the oxidant and reductant were added.

Slight copper elevation and up-regulation of MT1 and MT2 **(**which bind copper very tightly) raises the question of whether the modest copper misbalance has any negative consequences for cell metabolism. Copper is a redox-active metal, and it may alter the redox environment of intracellular compartments. Two main systems maintain thiol redox balance: a GSH/glutathione disulfide (GSSG) couple and a reduced/oxidized thioredoxin couple (31, 32). Conventional methods of measuring the GSH:GSSG ratio involve cell lysis and oxidation artifacts (33). Consequently, to characterize the redox status of cell compartments in patient cells, we used redox sensors and quantitative real-time imaging in live cells (Fig. 4).

For studies of the GSH:GSSG ratio in the cytosol, we used a recently developed GRX1-roGFP2 sensor (21, 34). To characterize the GSH:GSSG status in the nucleus, we added an SV-40 NLS to the N terminus of GRX1-roGFP2 (Fig. 4*A*). To target the sensor to mitochondria, we fused a triple MTS from subunit VIII of human cytochrome *c* oxidase to the GRX1-roGFP2 N terminus (Fig. 4*A*). This tag directed the sensor to the mitochondrial matrix. Correct localization of the cytosolic and nuclear sensors was apparent from the characteristic patterns of fluorescence (Fig. 4*B*). Targeting of MTS-GRX1-roGFP2 to mitochondria was confirmed by colocalization with MitoTracker Red (Fig. 4*B*).

The ability of sensors to identify changes in glutathione oxidation was verified by treatment of cells with DTT or H_2_O_2_. Oxidation/reduction of glutathione causes reciprocal changes in the excitation spectrum of GRX1-roGFP2 at two characteristic wavelengths (405 and 488 nm). The ratio of fluorescence intensities emitted following excitation at 405 and 488 nm (IR_405/488 nm_) increases when glutathione is oxidized and decreases upon treatment with a reductant (Fig. 4*C*). Each of the compartment-specific sensors showed a higher IR_405/488_ value in response to treatment of cells with 100 μm H_2_O_2_. The IR_405/488_ value was decreased upon subsequent treatment with 500 μm DTT (Fig. 4*C*).

**FIGURE 5.**
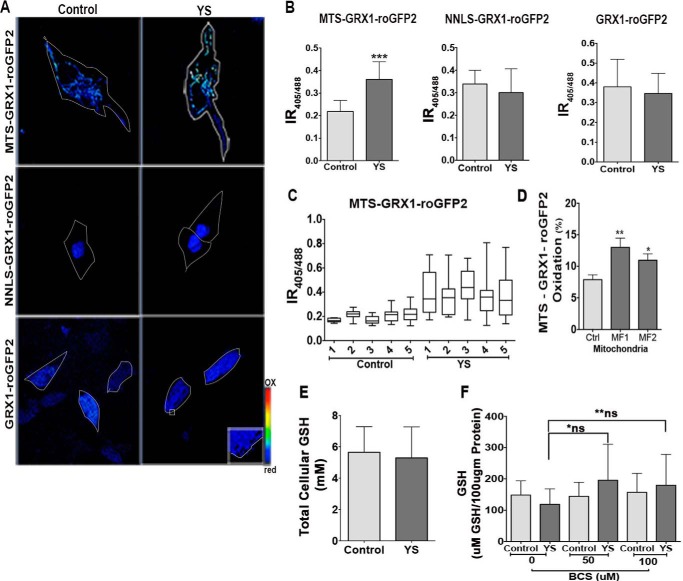
**Copper accumulation in YS cells is associated with increased oxidation of glutathione in mitochondria.** *A*, ratiometric false-colored image showing IR_405/488_ in YS and control cells for different compartments. YS cells have a higher IR_405/488_ value than control cells (0.36 ± 0.078 and 0.22 ± 0.049) in mitochondria (*MTS-GRX1-roGFP2*) and no change in the cytosol (*GRX1-roGFP2*) or nuclei (*NLS-GRX1-roGFP2*). Cells were imaged at ×40 magnification for GRX1-roGFP2 and NLS-GRX1-roGFP2 and at ×63 magnification for MTS-GRX1-roGFP2. *B*, average IR_405/488_ for different compartments in control and YS cells (***, *p* < 0.001). *C*, range of IR_405/488_ values determined by analysis of different regions of mitochondria within the same cell. *D*, average percent oxidation of MTS-GRX1-roGFP2 in control (*Ctrl*) and Menkes fibroblast (MF1 and MF2) cells (**, *p* = 0.006; *, *p* = 0.0231). *E*, GSH levels in control and YS cells. *F*, GSH level in mitochondria isolated from control and YS cells grown under basal conditions or treated with bathocuproine disulfonate. *, *p* = 0.3715; **, *p* = 0.6543; *ns*, not significant. Average data from five replicate experiments and 10 cells from each experiment are represented.

Patient cells showed increased glutathione oxidation, especially in mitochondria. We then used the sensors to characterize the status of glutathione pairs in different compartments of control and patient cells. In control cells, the IR_405/488_ values for the cytosol and nucleus were similar, indicating a similar oxidation state of the glutathione pair in these two compartments (Fig. 5, *A* and *B*). For mitochondria, IR_405/488_ was lower compared with the nucleus and cytosol, suggesting that glutathione was more reduced. This was unexpected because mitochondria are known to contain large amounts of peroxide. To verify our conclusion, we quantified fluorescence from ∼50 cells in each of the five independent experiments and obtained consistently low IR_405/488_ values (Fig. 5*C*). We also measured the IR_405/488_ value in different regions of mitochondria and found that, in control cells, the variation in sensor oxidation was small (Fig. 5*C*).

For patient cells, the oxidation state of glutathione in the cytosol and nuclei was similar, and the IR_405/488_ values did not differ significantly from the IR_405/488_ values of the corresponding compartments in control cells (Fig. 5*B*). Thus, copper accumulating in the cytosol and nucleus is efficiently buffered, and/or changes in glutathione oxidation are quickly corrected. In contrast, glutathione in YS mitochondria was significantly oxidized, as evident from a higher IR_405/488_ value (Fig. 5, *A* and *B*) compared with the control. Evaluation of various cells and different regions in mitochondria revealed that the maximum oxidation and variation in glutathione oxidation within the mitochondria were both significantly higher in the YS cells compared with the control (Fig. 5*C*). To verify that the observed differences in mitochondrial glutathione balance were not restricted to YS cells or were caused by genomic differences between YS and control cells, we examined the redox status of mitochondria using different control cells and two MD patient cell lines (MF1 and MF2). In both cases, MD mitochondria showed higher oxidation of the GRX1-roGFP2 sensor (Fig. 5*D*). Total levels of glutathione in cells and in isolated mitochondria fractions were similar for control and patient cells (Fig. 5, *E* and *F*). We attempted to test whether the change in the GSH:GSSG ratio in patient cells was caused by copper binding to GSH, which would make GSH unavailable for redox reactions. Treatment of mitochondrial homogenates with a copper chelator, bathocuproine disulfonate, on average increased available GSH; however, the increase was not statistically significant (Fig. 5*F*).

##### Glutathione Oxidation in Mitochondria Is Reversed by Expression of ATP7B

**FIGURE 6.**
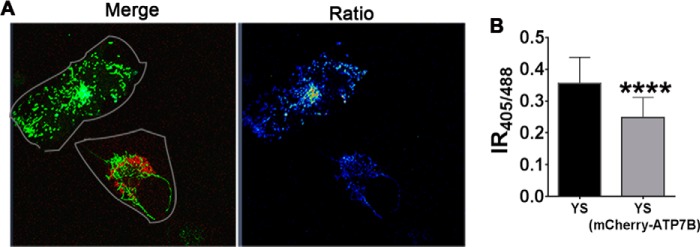
**Rescue of mitochondrial glutathione redox misbalance in YS cells by expression of ATP7B.** *A*, YS cells were co-transfected with MTS-GRX1-roGFP2 (*green*) and mCherry-ATP7B (*red*). The *merged image* shows cells expressing only MTS-GRX1-roGFP2 (*green*) and co-expressing MTS-GRX1-roGFP2 (*green*) and mCherry-ATP7B (*red*). The representative ratiometric image on the *right* shows that the IR_405/488_ values in an YS cell (0.36 ± 0.082) are higher than in neighboring YS cells expressing mCherry-ATP7B (0.25 ± 0.063). Cells expressing MTS-GRX1-roGFP2 have a higher ratio than cells expressing both proteins. *B*, average IR_405/488_ values in both cell types (****, *p* < 0.0001). Average data from three independent experiments with results from 10 cells/condition in each experiment are shown.

The mitochondrial matrix is thought to have a “copper storage pool” in the form of a complex with a small ligand, CuL, which apparently renders copper inert (35). We hypothesized that, in the absence of active ATP7A, copper that is normally transported into the secretory pathway enters the mitochondrial matrix, exceeding the buffering capacity of CuL and altering the GSH:GSSG balance. Restoration of copper flow through the secretory pathway should prevent the entry of unligated copper into mitochondria and restore the GSH:GSSG balance. To test this prediction, we co-transfected YS cells with MTS-GRX1-roGFP2 and mCherry-ATP7B (Fig. 6*A*, *left panel*). We used ATP7B instead of ATP7A for technical reasons. (The ability of ATP7B to substitute ATP7A in YS cells was demonstrated previously (17, 36–38)). In cells expressing ATP7B, the IR_405/488_ value was lower compared with cells lacking ATP7B (Fig. 6*A*, *right panel*), indicating that the restoration of the copper flux to the secretory pathway alone restored the mitochondrial glutathione balance, presumably by removing excess copper.

##### The Effect of ATP7A Inactivation on Mitochondrial Redox Balance Is Species-independent

**FIGURE 7.**
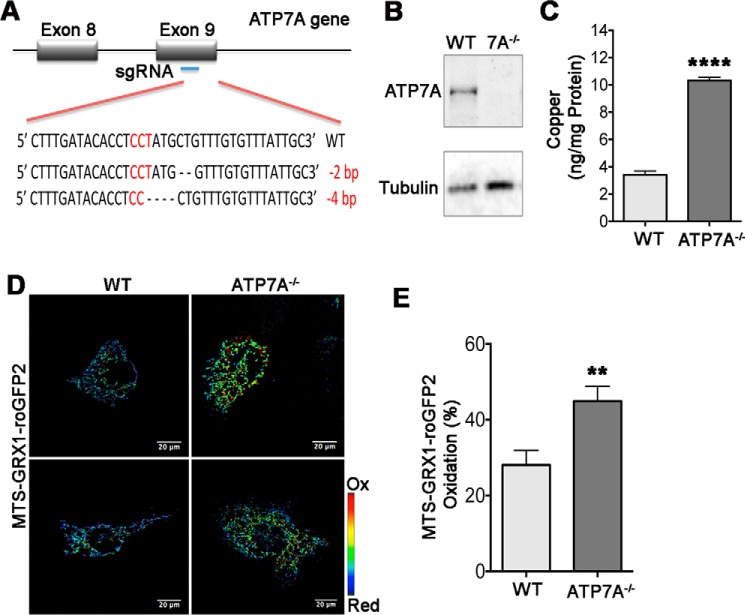
**Deletion of ATP7A in 3T3-L1 preadipocytes is associated with elevation of cellular copper content and increased oxidation of glutathione in mitochondria.** *A*, CRISPR/Cas9 targeting of exon 2 generated deletion in both alleles of genomic *ATP7A* in 3T3-L1 cells. *B*, Western blotting analysis of cell lysates shows the presence of ATP7A in the WT 3T3-L1 cells and a loss of ATP7A expression in CRISPR/Cas9-targeted 3T3-L1 cells. α-Tubulin was used as a loading control. *C*, copper levels in wild-type (3.4 ± 0.49 ng/mg protein) and ATP7A^−/−^ 3T3-L1 (10.32 ± 0.41 ng/mg protein) cells under basal conditions (****, *p* < 0.0001; *n* = 3). *D*, ratiometric (IR_405/488_) false-colored image of MTS-GRX1-roGFP2 fluorescence in WT and ATP7A^−/−^ 3T3-L1. Cells were imaged at ×40 magnification. *E*, oxidation of MTS-GRX1-roGFP2 in mitochondria of wild-type (28.08 ± 17.39, *n* = 20 cells) and ATP7A^−/−^ 3T3-L1 cells (44.90 ± 20.73, *n* = 28 cells). **, *p* = 0.0049. Data are mean ± S.E. of three independent experiments.

To test how general the effect of ATP7A on the GSH:GSSG pair in mitochondria is, we selected different cells (3T3-L1 preadipocytes, which express ATP7A but not ATP7B) from a different species (mouse) and inactivated ATP7A using the CRISPR/Cas9 system. The deletions in both alleles of the *ATP7A* gene were confirmed by sequencing (Fig. 7*A*). The mutation resulted in a frameshift and loss of ATP7A expression (Fig. 7*B*). Similar to human cells, the ability of ATP7A-deficient cells to proliferate in the basal medium was not negatively impacted. As expected, copper levels in ATP7A^−/−^ cells were elevated compared with control 3T3-L1 cells (Fig. 7*C*). Expression of the MTS-GRX1-roGFP2 sensor revealed higher sensor oxidation (higher IR_405/488_ value) in mitochondria of cells lacking ATP7A compared with control 3T3-L1 cells (Fig. 7, *D* and *E*), similarly to human ATP7A^−/−^ (YS) cells.

**FIGURE 8.**
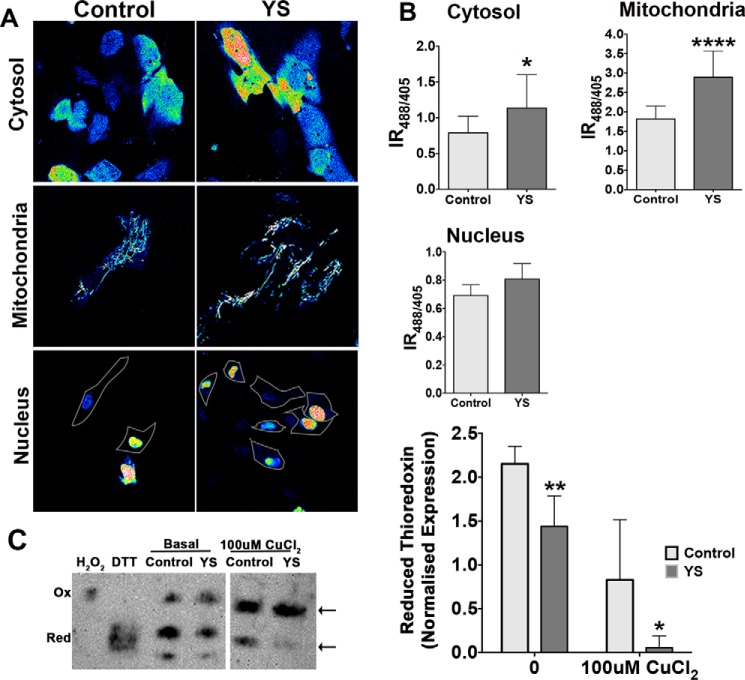
**High H_2_O_2_ and thioredoxin oxidation in the cellular compartments of YS cells.** *A*, representative ratiometric false-colored images of HyPer sensors targeted to different cellular compartments in control and YS cells. *B*, YS cells have higher IR_488/405_ values for mitochondrially targeted sensors compared with control cells (2.89 ± 0.67 and 1.82 ± 0.33, respectively) and for the sensor targeted to the cytosol (1.14 ± 0.47 and 0.79 ± 0.23, respectively). Data are mean ± S.D. from five independent experiments with 10 cells in each condition in each experiment (****, *p* < 0.0001; *, *p* = 0.0202). *C*, *left panel*, representative immunoblot of thioredoxin in control and YS cells grown under basal condition and either left untreated or treated with 100 μm CuCl_2_. *Arrows* indicate oxidized and reduced thioredoxin bands. Treatment of the mitochondria lysates with DTT or H_2_O_2_ was done to generate a fully reduced (*Red*) and oxidized (*Ox*) thioredoxin, respectively, to be used as a reference. *Right panel*, averaged normalized levels of reduced thioredoxin in control (2.15 ± 0.2) and YS cells (1.44 ± 0.35) under basal conditions and in response to treatment with 100 μm CuCl_2_ (control, 0.83 ± 0.62; YS, 0.06 ± 0.13). Data are mean ± S.D. (*n* = 3). **, *p* = 0.0177; *, *p* = 0.039.

H_2_O_2_ levels are higher in patient mitochondria. The above data indicated that accumulating copper altered the glutathione balance in the mitochondrial matrix, but the mechanism remained unclear. Elevated copper may directly affect (bind to or oxidize) glutathione, or it may increase the production of reactive oxygen species, resulting in accumulation of peroxide. To measure the levels of peroxide, we took advantage of genetically encoded ratiometric peroxide sensors (HyPer) targeted to the cytosol, nucleus, and mitochondria (22, 39). The IR_488/405_ ratio of fluorescence produced by the sensors is directly proportional to H_2_O_2_ levels. In control cells, peroxide was highest in mitochondria, as expected. The cytosol contained intermediate levels of peroxide, and the levels of H_2_O_2_ in nuclei were low (Fig. 8*A*). ATP7A inactivation and copper accumulation did not significantly change the peroxide content in the nucleus but increased cytosolic peroxide by 44.16%. The most significant increase (59%) was detected in mitochondria (Fig. 8*B*). We also found that a larger fraction of thioredoxin was oxidized in YS cells under basal conditions compared with the control and that Trx1 became fully oxidized when cells were treated with excess copper (Fig. 8*C*). Thus, both redox systems in MD patient mitochondria are compromised, and the levels of peroxide are higher than in the control.

##### Glutathione Oxidation in Mitochondria Is Independent of H_2_O_2_ Levels

**FIGURE 9.**
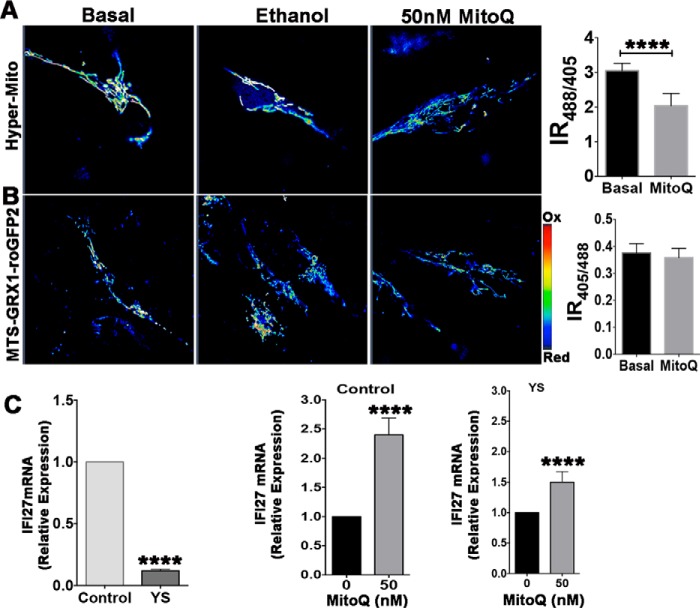
**MitoQ decreases the peroxide content in YS cells but does not reverse glutathione oxidation.** *A* and *B*, patient (*YS*) cells expressing the HyPer-Mito sensor (*A*) or the MTS-GRX1-roGFP2 sensor (*B*) were treated either with MitoQ or an equivalent volume of ethanol (vehicle). Ratiometric false color-coded images of sensor fluorescence are shown in the *left panels*. The *right panel* in *A* illustrates the decrease of the IR_488/405_ values of the Hyper sensor in response to MitoQ, indicative of decreased levels of peroxide. The *right panel* in *B* illustrates the lack of changes in the IR_405/488_ value for the MTS-GRX1-roGFP2 sensor; *i.e.* MitoQ treatment does not affect glutathione oxidation. The experiment was repeated three times with 25 cells analyzed in each experiment. *C*, relative IFI27 expression in YS and control cells measured by real-time PCR before and after treatment with MitoQ. ****, *p* < 0.0001. Data are mean ± S.D. (*n* = 3).

Copper can facilitate production of superoxide with consequent generation of peroxide, or it can oxidize GSH, decreasing the ability of GSH-dependent enzymes to eliminate H_2_O_2_. To discriminate between these possibilities, we used MitoQ, an antioxidant specifically targeted to mitochondria (40–42). Treatment of YS cells expressing HyPer-Mito with MitoQ decreased the IR_488/405_ value in YS mitochondria, indicative of lower H_2_O_2_ content compared with cells treated with an equivalent volume of ethanol (vehicle) (Fig. 9*A*). The decrease in oxidative burden by MitoQ was further demonstrated by measuring mRNA levels for IFI27, a mitochondrion-resident protein whose expression is influenced by oxidative stress (43). Treatment with MitoQ up-regulated IFI27 mRNA levels in both control cells and YS cells (Fig. 9*C*), consistent with the sequestration of peroxide. The treatment, however, did not change the status of the glutathione pair, as evidenced by an unchanged oxidation of MTS-GRX1-roGFP2 (Fig. 9*B*). Thus, although MitoQ diminishes mitochondrial H_2_O_2_, glutathione remains oxidized. This result suggests that elevation of H_2_O_2_ occurs downstream of glutathione and that glutathione is the primary (and/or peroxide-independent) target of elevated copper.

##### Redox Misbalance Markedly Sensitized Cells to a Decrease in Total Glutathione

**FIGURE 10.**
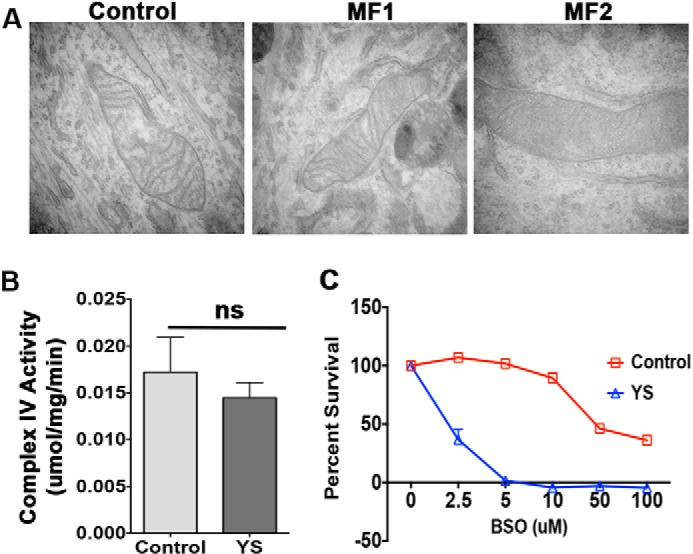
**MND patient fibroblasts are highly sensitive to glutathione depletion in the absence of significant changes in mitochondrial morphology and function.** *A*, electron microscopy of control and MND patient cells (MF1 and MF2) grown under basal conditions illustrates similar mitochondrial morphology. *B*, cytochrome *c* oxidase activity is similar in mitochondria freshly isolated from control and MND patient (*YS*) cells (*ns*, *p* = 0.2525; *n* = 3). *C*, inhibition of glutathione synthesis has markedly different effects on the viability of control and MND patient cells. YS and control cells were plated at 2000 cells/well density and treated with increasing concentrations of BSO (an inhibitor of glutathione synthase), washed, and stained with 3-(4,5-dimethylthiazol-2-yl)-5-(3-carboxy-methoxy-phenyl)-2-(4-sulfophenyl)-2H-tetrazolium. Data are mean ± S.D. (*n* = 3).

Oxidative stress may trigger mitochondrial fission and decrease activity of the respiratory chain. Consequently, to determine the functional consequences of redox misbalance in MD patient mitochondria, we characterized mitochondrial morphology and function. Electron microscopy of control and patient cells showed no significant differences in mitochondrion length, shape, or crista appearance (Fig. 10*A*). The activity of cytochrome *c* oxidase in freshly prepared mitochondria also did not differ significantly from the control (Fig 10*B*). We hypothesized that, under basal conditions, the mitochondria of ATP7A-deficient cells retain sufficient redox buffering capacity, which (given a negative effect of copper on GSH) would greatly depend on total available glutathione. To test this hypothesis, we treated the YS and control cells with different concentrations of the glutathione synthase inhibitor BSO (2.5–100 μm) to decrease the levels of cellular glutathione. Cell viability was quantified by 3-(4,5-dimethylthiazol-2-yl)-5-(3-carboxy-methoxy-phenyl)-2-(4-sulfophenyl)-2H-tetrazolium assay, which calculates mitochondrion functional integrity. The viability of control cells was unchanged in the presence of 2.5 μm BSO, whereas the number of live YS cells decreased to 37%. At higher inhibitor concentrations, YS cells were not viable, illustrating loss of their mitochondrial function upon decreasing glutathione levels.

## Discussion

ATP7A is the major copper transporter, with an established role in a dietary copper uptake and the delivery of copper to such important enzymes as dopamine-β-hydroxylase, peptidyl-α-mono-oxygenase, lysyl oxidase, SOD3, and others. Here we demonstrate that normal ATP7A transport activity is essential for maintaining the redox balance in mitochondria. This function is mediated through the sequestration of exchangeable “active” copper within the secretory pathway. The exchangeable copper, which enters the mitochondria when ATP7A is inactivated, decreases the ratio of reduced to oxidized glutathione and increases the levels of peroxide and protein oxidation. Copper may act by binding to GSH and thus lowering the amount of reduced glutathione, which in turn may decrease the activity of glutathione peroxidase and increase the levels of peroxide. The high sensitivity of MD cells to glutathione depletion and enhanced oxidation of thioredoxin are consistent with this scenario. More complex mechanisms could also be at play. In the liver, copper accumulation changes the activity of nuclear receptors, triggers changes in RNA splicing machinery, alters DNA methylation, and disregulates lipid metabolism (44–50). A systematic analysis of how redox misbalance contributes to the transcriptional, proteomic, and metabolic changes in cells and individual compartments would help to generate a comprehensive picture of the cellular response to copper overload.

The effects of copper in MD cells are observed under basal growth conditions when copper elevation is modest. This suggests that the copper-buffering capacity of the mitochondrial matrix is limited, and it is unlikely to function as a copper storage pool for the entire cell. The low molecular weight copper ligand CuL, which binds copper in the mitochondrial matrix, could be a specialized copper reservoir necessary for an uninterrupted biosynthesis of functional cytochrome *c* oxidase (35).

The observed marked effect of ATP7A inactivation on mitochondrial copper content and redox environment was somewhat unexpected because copper accumulation in MD fibroblasts has traditionally been considered mainly cytosolic. The sensitivity of mitochondria to high copper has been reported previously for Wilson disease and animal models of copper overload (48, 51, 52) and is thought to be an important component of disease pathology in copper overload states. The different sensitivity of the redox environment of cellular compartments to copper elevation raises many new questions. Does copper act differently in different cellular compartments, *i.e.* through binding to proteins/DNA in the nuclei and changes of redox buffers in mitochondria? Does redox misbalance in mitochondria yield metabolic changes that, in turn, trigger epigenetic changes in the nuclei? The response of which cellular compartment is more significant to cell adaptation to oxidative stress and cell survival? How are compartmental responses integrated? We speculate that mitochondria might be particularly susceptible to elevated copper because they lack dedicated protein chelators such as metallothioneins, and the transport/folding of proteins localized to the intermembrane space is mediated by the unique redox-dependent MIA (mitochondrial intermembrane space assembly) pathway.

To date, most efforts in developing therapies for Menkes disease have been directed toward increasing copper delivery to the CNS. Brain copper deficiency accounts for many of the neurological manifestations of classic Menkes disease. Our work adds a new dimension to a complex set of consequences associated with ATP7A inactivation. In addition to a well established loss of activity of copper-dependent enzymes in the secretory pathway, we demonstrate dysregulation of the redox-balancing system in mitochondria of ATP7A-deficient fibroblasts that hyperaccumulate copper because of impaired egress. The effect on the mitochondrial redox environment and, presumably, the function of various redox-dependent enzymes may be relevant in peripheral tissues of Menkes patients where copper accumulates (*e.g.* the kidney and intestine). Tissues and cells with a relatively low glutathione content or high demand for robust mitochondrial function (such as the heart and skeletal muscle) could also be adversely affected. Further clinical and laboratory investigations in animal models are warranted to better understand this phenomenon and its full implications.

## Author Contributions

M. R. and A. B. were involved in designing the experiments, data acquisition, data analysis and interpretation, and writing of the manuscript. S. L. was involved in designing the experiments, data analysis and interpretation, and writing of the manuscript. M. D., E. R., A. C. A., Y. W. L., H. Y., and T. C. were involved in data acquisition. L. B. was involved in data analysis and interpretation. M. P. M., L. Y., M. W., and S. G. K. provided critical reagents and read and commented on the manuscript.

## Footnotes

6 The abbreviations used are:

MD: Menkes disease
ICP: inductively coupled plasma
NLS: nuclear localization signal
MTS: mitochondrial target sequence
GSSG: glutathione disulfide
IR: intensity ratio
BSO: buthionine sulfoximine
CRISPR: clustered regularly interspaced short palindromic repeats.
